# Supplementary material for: Direct visualization of a Fe(IV)–OH intermediate in a heme enzyme
Source: Nat Commun. 2016 Nov 29;7:13445. doi: 10.1038/ncomms13445 (PMC5141285; doi:10.1038/ncomms13445)
Supplement: Supplementary Information — Supplementary Figures 1-6, Supplementary Table 1 and Supplementary References [file ncomms13445-s1.pdf]

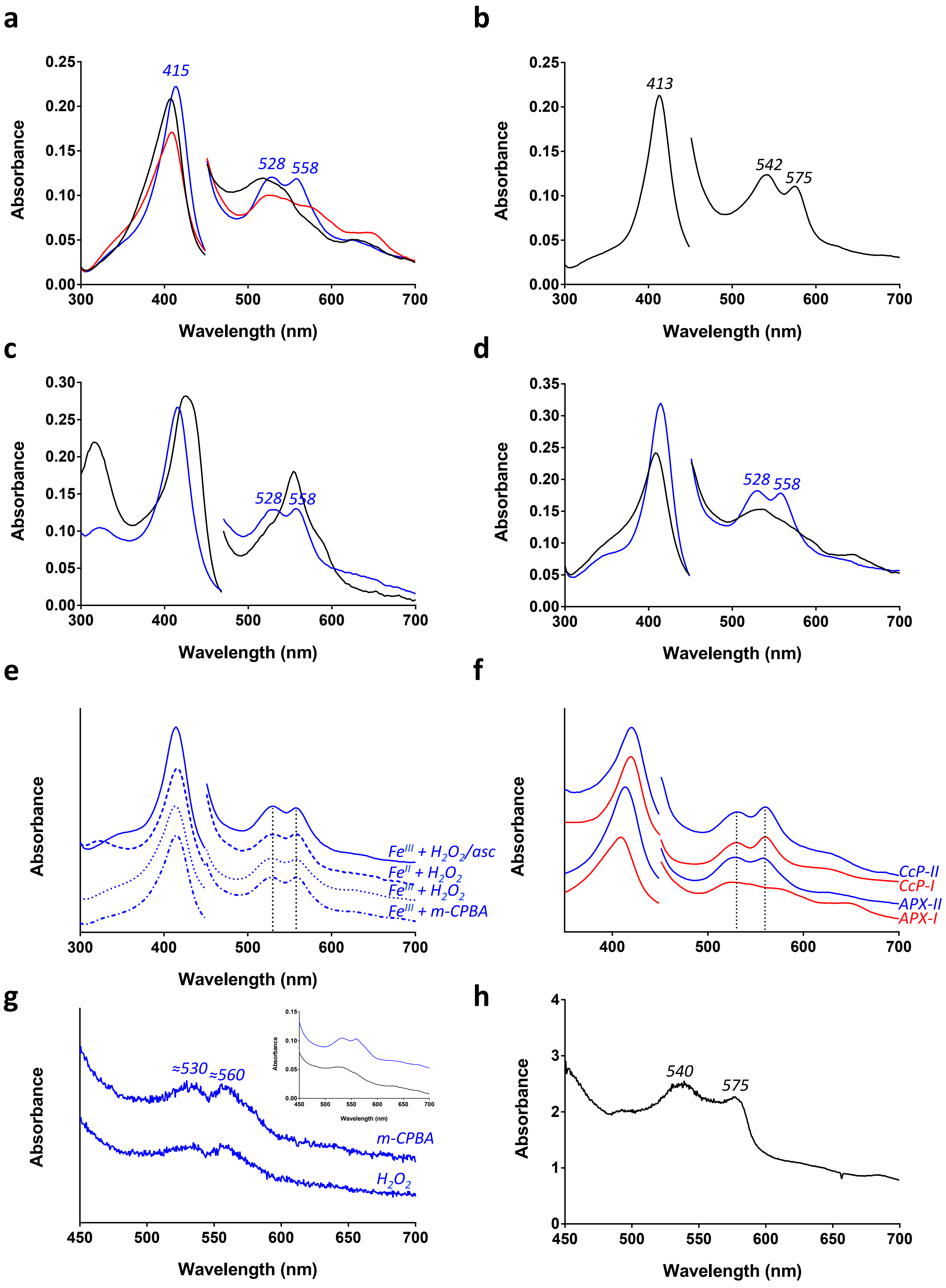

a
415
528
558
Absorbance
Wavelength (nm)
b
413
542
575
Absorbance
Wavelength (nm)
c
528
558
Absorbance
Wavelength (nm)
d
528
558
Absorbance
Wavelength (nm)
e
Fe^III + H2O2/asc
Fe^II + H2O2
Fe^III + H2O2
Fe^III + m-CPBA
Absorbance
Wavelength (nm)
f
CcP-II
CcP-I
APX-II
APX-I
Absorbance
g
≈530
≈560
m-CPBA
H2O2
Absorbance
Wavelength (nm)
h
540
575
Absorbance
Wavelength (nm)

**Supplementary Figure 1. Formation of Compound II.** In all panels, Compound I intermediates are in red, and Compound II intermediates are in blue.

**a,** Stopped flow UV-visible spectra obtained on reaction of ferric APX (black line, at t = 0) with $H_2O_2$ (20 equivalents), monitored over 100s, showing formation of Compound I (red line, $\lambda_{max}$ 409, 527, $575^{sh}$, 649 nm) and then Compound II (blue line, $\lambda_{max}$ = 415, 528, 558 nm). These spectra are identical to those presented in Fig. 1a. Compound II spectra from tea APX ($\lambda_{max}$ = 419, 530, 558 nm[1]) and pea APX ($\lambda_{max}$ = 414, 528, 559 nm[2]; $\lambda_{max}$ = 413, 528, 558 nm[3]) have been reported with very similar maxima. With $H_2O_2$, formation of Compound III (by reaction of Compound II with excess $H_2O_2$, Fig. S1b) is a complicating issue, and is avoided by using *m*-CPBA. Compound II prepared using other methods (Figs. 1c-e) give very similar spectra, which demonstrates that Compound II species prepared by different methods is the same.
**b,** UV-visible spectrum of the non-catalytic Compound III formed on reaction of ferric APX with 200 equivalents of $H_2O_2$. Formation of Compound III (by reaction of Compound II with excess $H_2O_2$) is avoided with *m*-CPBA and was thus used in the crystallography experiments.
**c,** Stopped flow UV-visible spectra obtained on reaction of ferrous APX (black line, formed by reaction of ferric enzyme with dithionite) with 10 equivalents of $H_2O_2$ under anaerobic conditions, showing formation of Compound II (blue line, $\lambda_{max}$ = 416, 528, 558 nm). This spectrum is the same as that observed in **a**, and in Fig. 1a.
**d,** Stopped flow spectra obtained on reaction of ferric APX (black line, incubated with 1.5 equivalents of ascorbate) with 1.5 equivalents of $H_2O_2$, showing formation of Compound II (blue line, $\lambda_{max}$ = 414, 528, 558 nm). This spectrum is the same as those observed in **a** and **c**, and in Fig. 1a.
**e,** Overlay of Compound II intermediates obtained from **a**, **c** and **d** with that formed by reaction of ferric APX with *m*-CPBA (Fig. 1a), showing the similarity of all the spectra. From bottom spectrum upwards: dash-dot line = ferric APX + *m*-CPBA from Fig. 1; dotted line = ferric APX + $H_2O_2$ from **a**; dashed line = ferrous APX + $H_2O_2$ from **c** above; solid line = ferric with $H_2O_2$/ascorbate from **d**. Spectra have been offset on the y-axis, for clarity. A comparison with C*c*P spectra is presented in 1f.
**f,** Comparison of the Compound I (red line) and Compound II (blue line, $\alpha/\beta$ = 0.97) spectra obtained in this work (from Fig. 1a) with that of C*c*P-I (red line, formed on reaction of ferric CcP with 1.5 equivalents of $H_2O_2$, $\alpha/\beta$ = 1.11) and C*c*P-II (blue line, formed directly on reaction of ferrous C*c*P with 15 equivalents of $H_2O_2$, $\alpha/\beta$ = 1.10). The $\alpha/\beta$ bands are suggested to report on protonation state[4], with the $\alpha$-band decreasing in intensity on formation of Fe(IV)-OH. Our spectra are consistent with this interpretation. Spectra have been offset on the y-axis, for clarity.
**g,** Comparison of single crystal UV-visible spectra (100 K) of Compound II formed by reaction with *m*-CPBA (top spectrum, taken from Fig. 1a inset) with that of a single crystal reacted with $H_2O_2$ (bottom spectrum). Both spectra show the characteristic Compound II peaks ($\lambda_{max}$ = 530 and 560 nm) in the visible region. Note that different crystals of Compound II, formed in the same way by reaction of a ferric crystal with *m*-CPBA or $H_2O_2$, and then re-dissolved in buffer (10 mM potassium phosphate pH 7.0, 150 mM KCl) give maxima that agree with those in solution (not shown). The inset shows the spectrum of crystals of APX that had been reacted with *m*-CPBA to give Compound II (blue line) and then redissolved in buffer as above, showing that the spectrum reverts back to a ferric species (black line) as expected (and on the same timescale as is reported[5] in solution). The spectra have been offset on the y-axis, for clarity. The maxima for Compound II in these spectra are different from those for single crystals of ferric APX at 100 K ($\lambda_{max}$ = 540, 575 nm, spectrum **h** below; we assign the spectrum of ferric APX at 100 K to formation of a low-spin Fe(III)-OH species, by analogy with known low-spin heme-hydroxide species[6].
**h,** Single crystal UV-visible spectrum of ferric APX at 100 K showing maxima at 540 and 575 nm. These maxima are different from those of Compound II.

For **a** – **f**, absorbance values in the visible region have been multiplied by a factor of four. Conditions for **a** – **d**: 10 mM sodium phosphate, 150 mM KCl pH 7.0, 10.0 °C

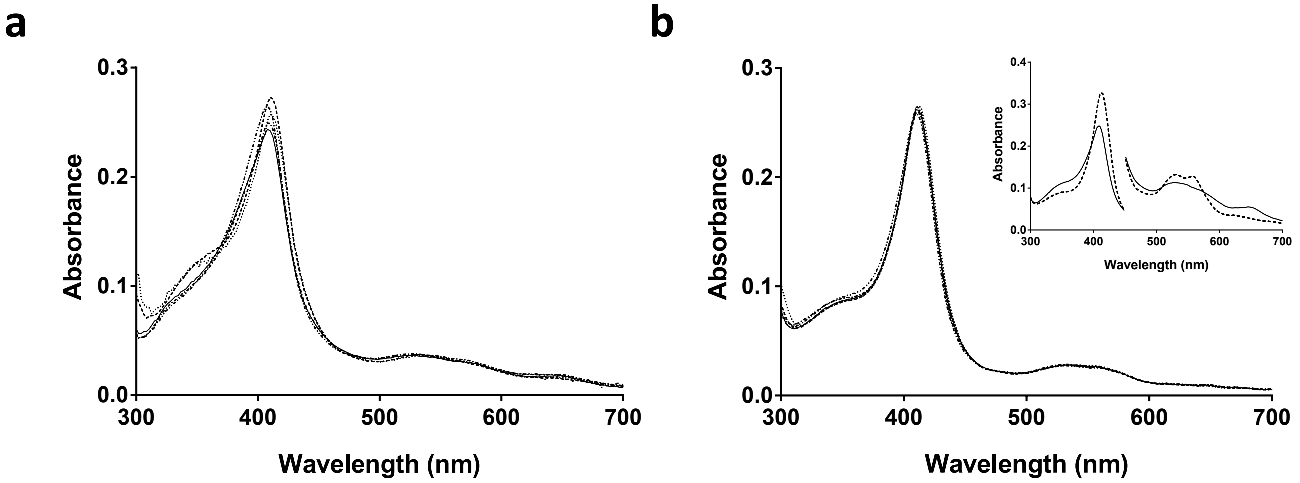


**Supplementary Figure 2. pH dependence of Compound I and Compound II spectra. a,** Absorbance spectra of Compound I as a function of pH. Spectra were obtained in a single-mixing stopped-flow experiment by reaction of APX (prepared in water, adjusted to pH 7) with 0.2 M buffer at the desired final pH (see main text for details). Outside of the pH range 4.5 – 11.5 the enzyme was not stable. **b,** Absorbance spectra of Compound II as a function of pH. Spectra were obtained in a double-mixing stopped-flow experiment. Ferric APX was first mixed with 10 equivalents of $H_2O_2$ (both in water, pH 7.0) and incubated for 80 s to allow maximum formation of Compound II. This solution was then mixed with 0.2 M buffer to obtain the desired final pH. Inset: formation of Compound II at pH 7 (prior to the pH jump); solid line = Compound I, dashed line = Compound II. In **a** and **b**, higher pH ranges were not accessible due to instability of the enzyme on the stopped-flow timescale at pH > 11.5. In **a** and **b**, spectra are as follows: pH 11.5 (dotted line), pH 10.5 (dashed line), pH 9.0 (solid line), pH 7.0 (dot-dashed line) and pH 4.5 (dot-dot-dashed line).

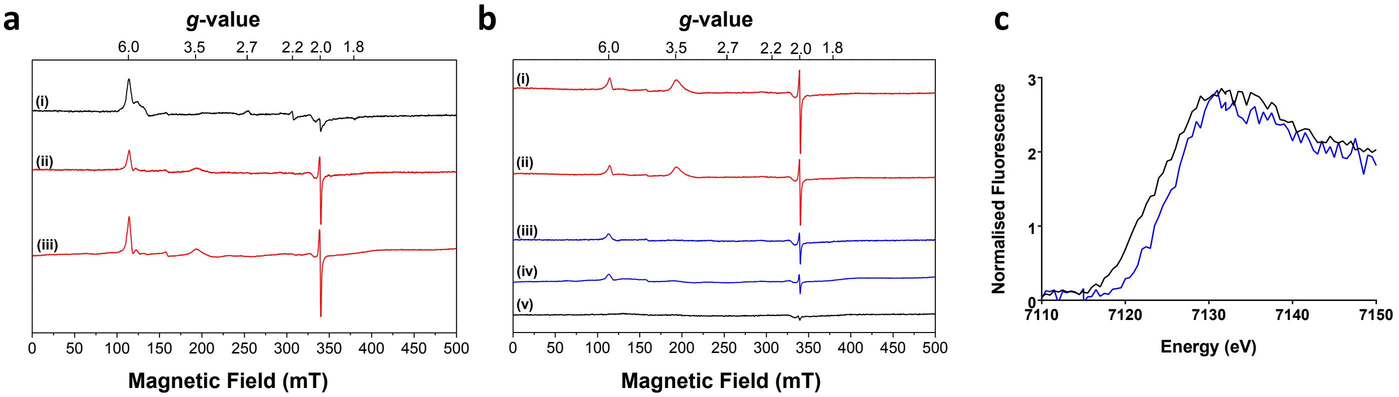


**Supplementary Figure 3. EPR and X-ray fluorescence analyses. a,** Formation of Compound I on reaction of ferric APX with *m*-CPBA. 9 GHz EPR spectra of a solution of: (**i**) Ferric APX; (**ii**) Compound I formed by reaction of ferric APX with 1-equivalent of *m*-CPBA and flash frozen 7s after mixing. A decrease in intensity of the high- and low-spin ferric signals is observed, and formation of a new species at g = 3.54. (**iii**) The same Compound I sample as (ii) but recorded after 20 days. **b,** Formation of Compound I and Compound II on reaction of ferric APX with $H_2O_2$. **(i)** Compound I formed formed by reaction of ferric APX with 1-equivalent of $H_2O_2$ and flash frozen 7s after mixing; (**ii**) The same sample as (i) but recorded after 20 days; (**iii**) Compound II prepared by reaction of ferric APX with 20-equivalents of $H_2O_2$ and flash-frozen after 40s; experiments with a greater excess of $H_2O_2$ showed no new resonances, consistent with the formation of EPR-silent, Compound III. (**iv**) the same sample as (iii) but recorded after 20 days; (**v**) the spectrum of buffer (10 mM potassium phosphate buffer, 150 mM KCl, pH 7.0), showing the cavity background. **c**, X-ray fluorescence spectra of single crystals of ferric APX (black) and Compound II (blue). Crystals were scanned between 7110 and 7150 eV. The spectrum shows the expected shift to higher energy in Compound II, consistent with the presence of a higher oxidation state of the iron.

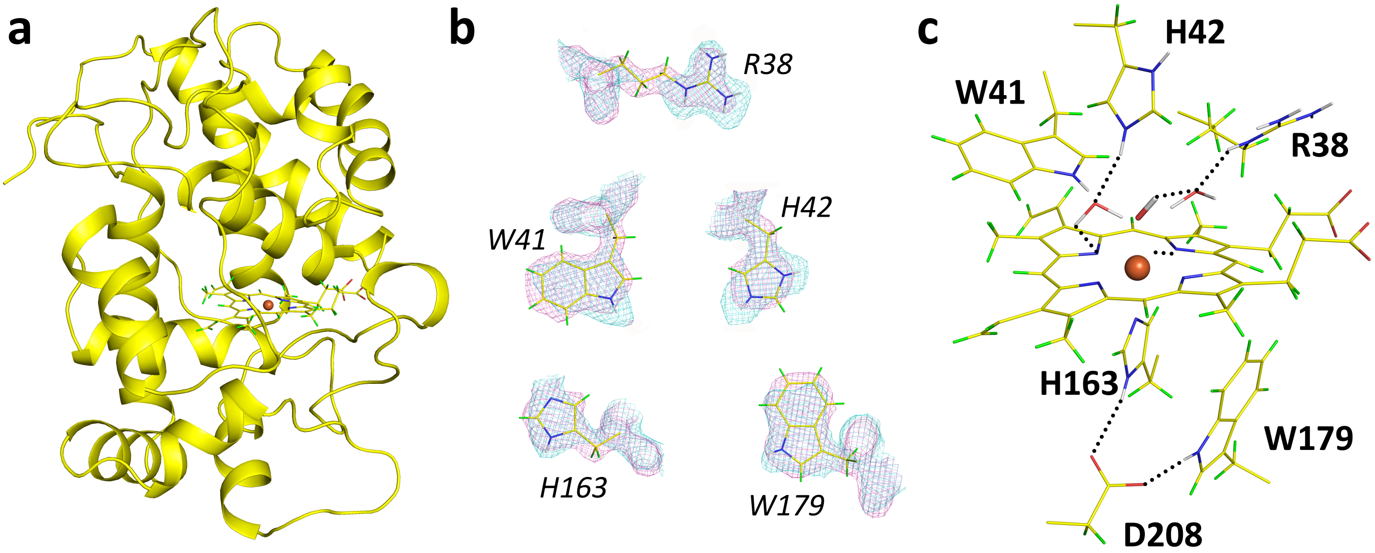


**Supplementary Figure 4. Detailed view of heme active site residues in Compound II. a**, The overall neutron structure of APX. The heme is shown in stick representation. **b,** Nuclear (cyan) and electron (magenta) scattering densities of the active side residues observed in Compound II. All maps are contoured at 1.5 σ. The diagram presents: Arg38, showing deuteration of all nitrogen atoms of the guanidinium group; Trp41, showing deuteration of Nε; His42, showing protonation of both Nδ and Nε; His163, showing deuteration of Nε (Nδ is coordinated to the iron (not shown)); Trp179, showing deuteration of Nε. Hydrogen atoms are in green and deuterium atoms are in white. **c,** Water molecules and hydrogen bonding network in the distal heme pocket. The distal pocket has two water molecules. Colour scheme: hydrogen - green; deuterium - white; carbon - yellow; oxygen - red; nitrogen - blue; iron - brown sphere. For clarity, not all hydrogen bonds are shown.

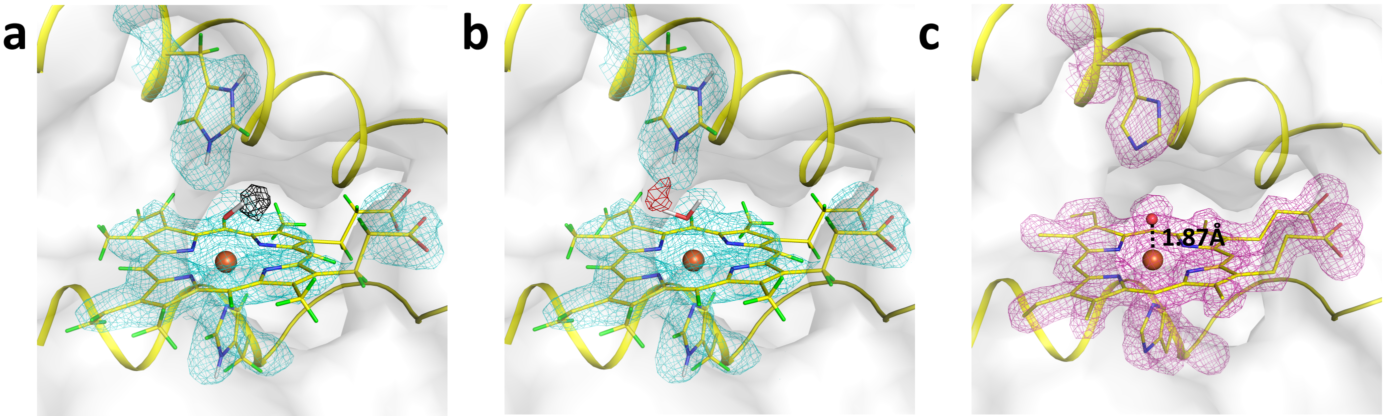


**Supplementary Figure 5. Validation of deuterium position and Fe-O bond length in Compound II. a,** Nuclear difference density map (shown in black, contoured at 2.6 $\sigma$) calculated using oxygen at the distal position; a positive peak at the deuterium atom position is observed. The nuclear 2Fo-Fc map is shown in cyan (contoured at 1.5 $\sigma$). **b,** Nuclear difference density map (shown in red, contoured at 2.8 $\sigma$) calculated using $D_2O$ at the distal position. A negative peak is observed. **c**, Multi-crystal X-ray structure of Compound II confirms the Fe-O bond length. The structure was solved by merging the first 10° of data from 10 different crystals. The Fe-O distance is 1.87 Å. The electron density map in magenta was contoured at 1.5 $\sigma$. The ferryl oxygen atom is shown as a red sphere and iron is shown as a brown sphere.

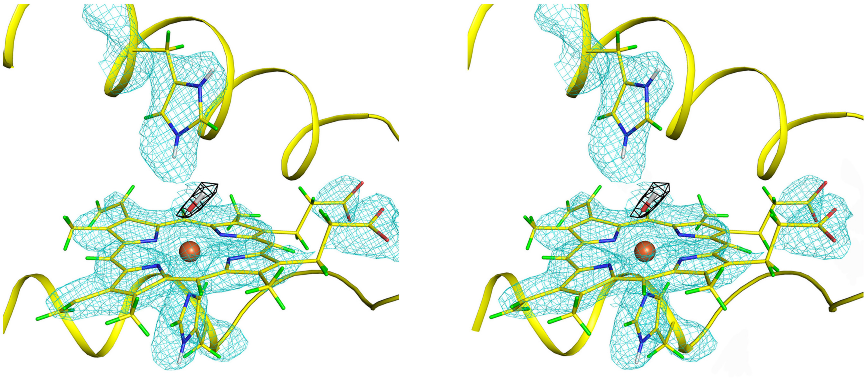

**Supplementary Figure 6. Stereo image of nuclear scattering density in the crystal structure of Compound II.** Nuclear scattering density is shown in cyan (contoured at 1.5 $\sigma$). The neutron Fo-Fc difference density calculated by omitting the distal ligand is shown in black (contoured at 3.0 $\sigma$). Colour scheme: hydrogen - green; deuterium - white; carbon - yellow; oxygen - red; nitrogen - blue; iron - brown sphere. This is the same view as Figure 2c.

## Supplementary Table 1

Data collection and joint X-ray/neutron structural refinement statistics for APX-II.

| | |
|---|---|
| Space group | $P4_22_12$ |
| Cell dimensions (Å) | 82.095, 82.095, 75.162 |
| **Data collection (Neutron, quasi-Laue λ = 3.2 – 4.2 Å)** | |
| Resolution | 40.00 - 2.31 (2.43 – 2.31) |
| $R_{merge}$ | 0.18 (0.21) |
| I/σI | 7.1 (4.7) |
| Completeness (%) | 73.1 (50.0) |
| Redundancy | 4.1 (2.4) |
| **Data collection (X-ray λ = 1.5418 Å)** | |
| Resolution | 19.91 – 1.80 (1.84 – 1.81) |
| $R_{merge}$ | 0.16 (0.69) |
| I/σI | 9.4 (2.1) |
| Completeness (%) | 98.72 (92.0) |
| Redundancy | 6.8 (4.0) |
| **Refinement** | |
| $d_{min}$ (Neutron, Å) | 2.31 |
| $d_{min}$ (X-ray, Å) | 1.81 |
| No. of reflections (Neutron) | 8367 |
| No. of reflections (X-ray) | 23856 |
| $R_{work}/R_{free}$ (Neutron) | 0.20/0.27 |
| $R_{work}/R_{free}$ (X-ray) | 0.16/0.21 |
| RMSD | |
| Bond length (Å) | 0.01 |
| Bond angle (°) | 1.35 |

## Supplementary References


1 Kvaratskhelia, M., Winkel, C., Naldrett, M. T. & Thorneley, R. N. F. A novel high activity cationic ascorbate peroxidase from tea (Camellia sinensis) - A class III peroxidase with unusual substrate specificity. *J Plant Physiol* **154**, 273-282 (1999).

2 Lad, L., Mewies, M. & Raven, E. L. Substrate binding and catalytic mechanism in ascorbate peroxidase: evidence for two ascorbate binding sites. *Biochemistry* **41**, 13774-13781 (2002)

3 Marquez, L. A., Quitoriano, M., Zilinskas, B. A. & Dunford, H. B. Kinetic and spectral properties of pea cytosolic ascorbate peroxidase. *FEBS letters* **389**, 153-156 (1996).

4 Horner, O. *et al.* Spectroscopic description of an unusual protonated ferryl species in the catalase from Proteus mirabilis and density functional theory calculations on related models. Consequences for the ferryl protonation state in catalase, peroxidase and chloroperoxidase. *J Biol Inorg Chem* **12**, 509-525 (2007).

5 Hiner, A. N. P. *et al.* Detection of a radical intermediate in the reaction of ascorbate peroxidase with hydrogen peroxide. *European Journal of Biochemistry* **268**, 3091-3098 (2001)

6 Antonini, M. & Brunori, E. *Hemoglobin and Myoglobin and their Reactions with Ligands*. (North Holland Publishers, 1971).
